# miR-26a-5p is a Stable Reference Gene for miRNA Studies in Chondrocytes from Developing Human Cartilage

**DOI:** 10.3390/cells8060631

**Published:** 2019-06-22

**Authors:** Enrico Ragni, Paola De Luca, Antongiulio Marmotti, Laura de Girolamo

**Affiliations:** 1 IRCCS Istituto Ortopedico Galeazzi, Laboratorio di Biotecnologie Applicate all’Ortopedia, I-20161 Milano, Italy; deluca.paola@grupposandonato.it (P.D.L.); laura.degirolamo@grupposandonato.it (L.d.G.); 2 Department of Orthopaedics and Traumatology, University of Turin, I-10124 Torino, Italy; antonio.marmotti@inwind.it

**Keywords:** miRNA, cartilage, reference gene, qRT-PCR, development, chondrocyte

## Abstract

miRNAs are emerging as key regulators of complex biological systems in several developmental processes. qRT-PCR is a powerful tool to quantitatively assess the profiles and modulation of miRNA expression. In the emerging field of cartilage maturation studies, from precursor to hypertrophic chondrocytes, few data about miRNA regulation are available, and no consensus on the best reference gene (RG) has been reached. This is a crucial pitfall since reliable outcomes depend on proper data normalization. The aim of this work was to identify reliable and stable miRNA RGs, basing the analysis on available high throughput qRT-PCR miRNA data (from the NCBI Gene Expression Omnibus database, GSE49152) obtained from human embryonic cartilage tissues enriched in the precursor, differentiated, and hypertrophic chondrocytes. Four normalization approaches were used, and the stability was quantified by combining BestKeeper, delta-Ct, geNorm, and NormFinder statistical tools. An integrated approach allowed to identify miR-26a-5p as the most stable RG and miR-212-3p as the worst one. RNU44, used in original dataset analysis, performed as second best RG. Applications of different normalization strategies significantly impacted the profiles and modulation of miRNA expression. Herein presented results point out the crucial need of a consensus on data normalization studies aimed at dissecting miRNA role in human cartilage development, to avoid the postulation of unreliable biological conclusions.

## 1. Introduction

Gene expression regulation is the biological foundation for the specification of every cell type, tissue, and organ in a multicellular organism. Together with transcriptional regulators, miRNAs have emerged as gene expression repressors directing the post-transcriptional modulation that underlies development [1]. miRNAs are evolutionary conserved, single-stranded, 21–24 nucleotide-long non-coding RNA molecules [2]. They are usually transcribed from DNA sequences first into primary miRNAs, then processed into precursor miRNAs and eventually mature molecules. The main mechanism of action is through direct interaction with 3′ untranslated region (3’ UTR) of target mRNAs, which induces mRNA degradation resulting in translational repression [3]. In addition, miRNAs may interact with 5′ UTR, coding sequence, gene promoters, and under certain conditions, they can also activate translation or regulate transcription [2]. To date, 2300 validated human miRNAs have been reported, and new miRNAs are still being discovered with their roles in gene regulation, starting to be fully deciphered [4].

miRNAs are involved in a variety of biological processes regulating human development in different districts. For some of them, a clear picture has been depicted [5]. As a striking example, for neural development, hundreds of miRNAs are involved in determining the fate of the two major cell types of the nervous system, neurons and glia, at both embryonic and early postnatal stages [6]. An advantage of the neural system is that these processes of neuro- and gliogenesis involve many intermediate cell types that have been exhaustively studied in terms of gene expression and non-coding RNAs regulation [7]. Similarly, in the bone system, the precise roles of many miRNAs have been fully deciphered, giving valuable insights into the treatment of developmental disorders of the skeleton [8]. On the contrary, despite an increasing amount of in vitro data, there is still limited information on the expression and function of specific miRNAs in human cartilage development in vivo.

During embryonic development, mesenchymal progenitor cells differentiate into chondrocytes to form cartilage templates for future bones and cartilage. Proliferating chondrocytes produce extracellular matrix, enriched in type II collagen and aggrecan. Further, they differentiate into hypertrophic chondrocytes that express type X collagen and undergo mineralization to be eventually replaced by mineralized bone, leading to longitudinal bone growth [9]. In this process, different signaling molecules and transcription factors have been shown to regulate the progression of chondrocyte-specific gene expression [10,11]. Regarding miRNAs, few data are available in the in vivo settings, and actual knowledge has predominantly come from studies in mice. let-7 miRNA is required for chondrocyte proliferation [12], whereas miR-140 modulates premature hypertrophic chondrocyte differentiation and delays differentiation of resting chondrocytes to proliferating chondrocytes [13,14]. Nevertheless, data of the human model depicting modulations or differences between cells at different stages of differentiation were missing.

To overcome the limitations of these studies, a pivotal work on miRNA expression patterns was performed by high-throughput qRT-PCR technique within human embryonic (gestational day 54–56) cartilage laser dissected fragments, containing either precursor (PC), differentiated (DC), or hypertrophic (HYP) chondrocytes (Gene Expression Omnibus database, GSE49152) [15]. This report was selected being unique in combining miRNA analysis and specific chondrocyte populations directly involved in regulating cartilage and long bone development. Authors were able to identify differentially expressed miRNAs predicted to regulate growth factors (vascular endothelial growth factor (VEGF), insulin-like growth factor-1 (IGF-1), transforming growth factor beta (TGF-β), bone morphogenetic protein (BMP), fibroblast growth factor (FGF)), Hedgehog and Wnt signaling pathways, and interleukin (IL)-8. Despite this fundamental milestone, only around 50 miRNAs were detectable in all the nine donors, leaving room for further studies aimed either at identifying those candidates that were missed for both technical or low expression reasons, or at studying dysregulation of miRNAs modulating developmental processes involved in skeletal disorders, such as osteoarthritis (OA), chondrodysplasias, or delayed endochondral fracture healing.

For a deep analysis of single or few miRNAs, a robust normalization strategy is a fundamental requisite. Therefore, mining qRT-PCR data of McAlinden dataset, through gold-standard statistical tools (BestKeeper [16], geNorm [17], NormFinder [18], and the comparative delta-Ct method [19]), the aim of this work was to identify stable miRNAs to be used as Reference Genes (RGs) in future studies dissecting these small RNAs involved in cartilage developmental or pathologic processes.

## 2. Results

### 2.1. miRNA Selection

**Figure 1:**
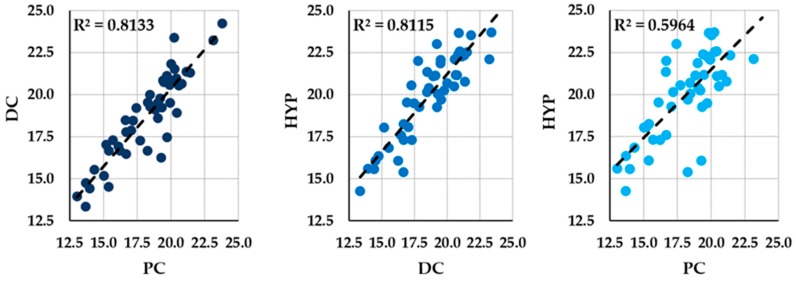
Pearson correlation scatter plots for the 46 miRNAs and RNU44 amplified from precursor (PC), differentiated (DC), and hypertrophic (HYP) tissues. x and y-axis indicate Ct values. R2 stands for the correlation coefficient, with values >0.8 meaning a very strong interrelationship and value <0.6 a fair one.

Mining qRT-PCR data, it was possible to identify 46 miRNAs with positive amplification values in all the 26 samples (8 PC, 9 DC, and 9 HYP). RNU44, used in McAlinden work as normalizer, was also always scored. The distribution of the quantification cycles (Ct) values of the selected reference genes over the whole sample sets is shown in Appendix A Table A1. miRNAs presented different expression values and variability levels in the three datasets. In PC chondrocytes, miR-26a-5p showed the largest expression (mean Ct = 13.04), while miR-362-3p was the least expressed (mean Ct = 23.80). In DC samples, miR-720 (mean Ct = 13.34) and miR-362-3p (mean Ct = 24.24) were poles apart. In HYP tissues, miR-720 (mean Ct = 14.26) was the most abundant, with miR-362-3p being again low expressed (mean Ct = 25.83). RNU44 was scored with low Ct values (mean Ct = 13.97 for PC, 14.42 for DC, and 15.57 for HYP). In terms of variability, miR-769-5p in PC, miR-202-3p in DC and HYP displayed the lowest standard deviation (SD), 0.51, 1.02, and 1.48, respectively. Eventually, correlation analysis was performed between each dataset. The analysis, shown in Figure 1, pointed out the existence of high correlation (R^2^ > 0.8) for PC/DC and DC/HYP when compared to one another, while PC and HYP had lower interrelationship (R^2^ = 0.6). This is in agreement with modulation of miRNA expression between precursor and hypertrophic chondrocytes, passing by an intermediate differentiation phase.

### 2.2. Candidate miRNA Ranking

**Figure 2:**
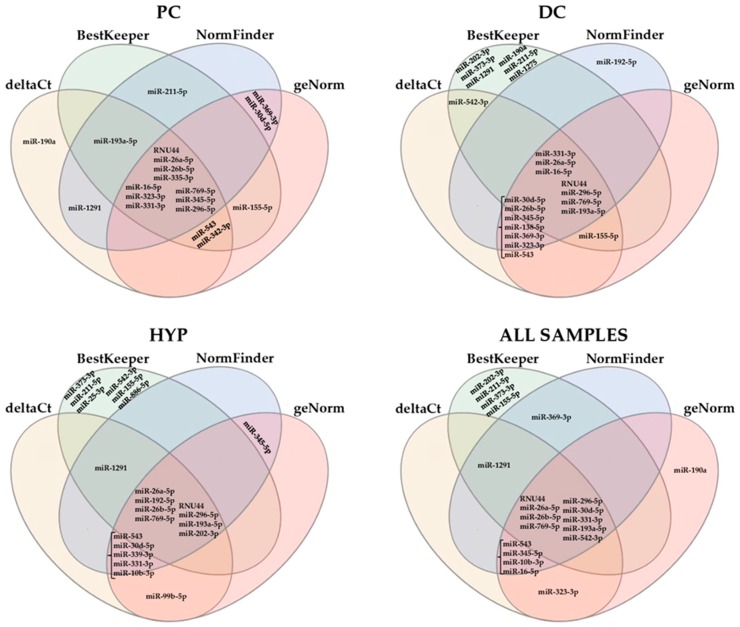
Venn diagrams of the overlap of proposed miRNA reference genes (RGs) through the four algorithms. The most stable 15 miRNAs per each algorithm were considered. PC: precursor; DC: differentiated; HYP: hypertrophic.

We picked the 46 miRNAs, together with RNU44, and assessed their potential contribution as normalizers following BestKeeper, geNorm, and NormFinder applets and comparative delta-Ct method (Appendix A Table A4, Table A5, Table A6 and Table A7) and Figure 2 for the first fifteen miRNAs in the different rankings). BestKeeper was first used to calculate the Ct standard deviation (SD) in the three datasets. This application ranked: in PC, miR-769-5p (SD of 0.38) and miR-212-3p (5.25) as the best and worst performers, respectively; in DC, miR-202-3p (0.64) and miR-212-3p (3.42); in HYP, miR-373-3p (1.14) and miR-199a-3p (5.03). Based on fold change data, geNorm generated a stability value M by stepwise exclusion of the candidate genes: in PC, miR-296-5p/miR-331-3p as the most stable pair of miRNAs (M of 0.25) and miR-99b-5p (3.37) as the worst RG; in DC, miR-16-5p/miR-26a-5p (0.30) and miR-119a-3p (2.28); in HYP, miR-331-3p/miR-10b-3p (0.51) and miR-520b (3.31). The comparative delta-Ct method identified: in PC, RNU44 (SD of 2.40) and miR-99b-5p (6.85) as best and worst RGs; in DC, miR-331-3p (1.61) and miR-199a-3p (4.22); in HYP, miR-26a-5p (2.33) and miR-520b (6.63). Eventually, NormFinder ranked: in PC, miR-26b-5p (0.45) as the most accurate and miR-99b-5p (6.46) as the less reliable RG; in DC, miR-331-3p (0.18) and miR-199a-3p (4.08); in HYP, miR-26a-5p (0.27) and miR-520b (6.21). Further, because the four applets generated different rankings, a comprehensive stability value was generated (geomean). Following this computation, RNU44 (2.40) for PC, miR-331-3p (2.63) for DC, and miR-26a-5p (2.89) for HYP samples were proposed as the most stable RGs.

Since both single algorithms and overall ranking resulted in different reliable RGs for each dataset, all samples were scored together. In this condition, BestKeeper suggested miR-202-3p (1.04), geNorm miR-26a-5p/miR-331-3p couple (0.58), delta-Ct method miR-26a-5p (2.30), and NormFinder miR-26a-5p (0.56) as the most accurate RGs. The comprehensive ranking of gene stability obtained by combining the four analyses (geomean) assessed miR-26a-5p (1.86) as the most stable miRNA, followed by RNU44 (3.31) and miR-26b-5p (3.98). miR-212-3p was the least stable candidate.

### 2.3. Impact of Normalization Strategy on miRNA Profiling

**Figure 3:**
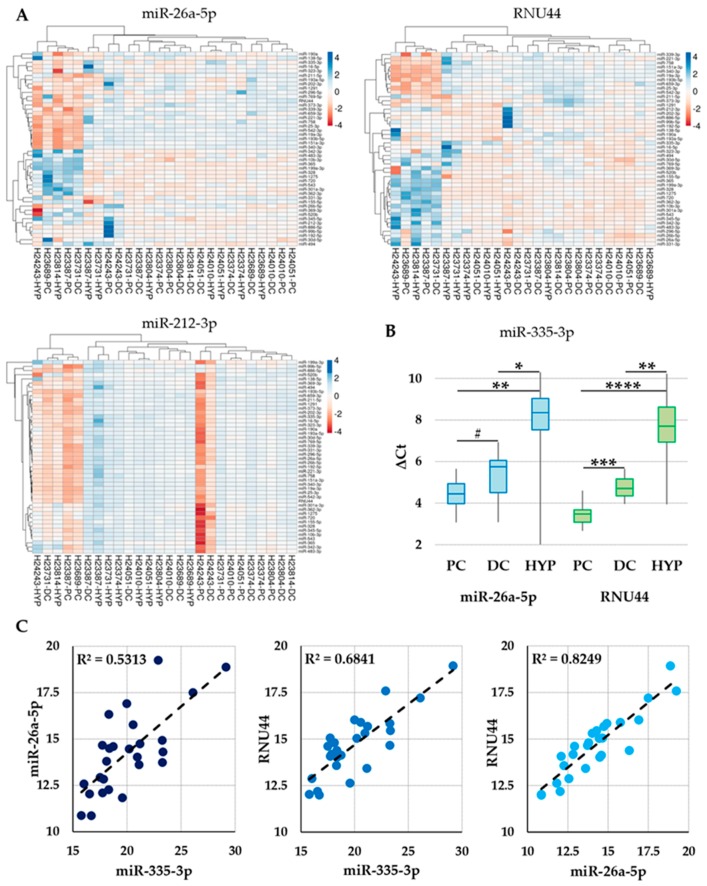
Effect of normalization strategy on the miRNAs scored in the precursor (PC), differentiated (DC), and hypertrophic (HYP) tissues. (**A**) Heat maps of the assayed miRNAs across all samples after miR-26a-5p, RNU44, or miR-212-3p normalization. (**B**) Effect of miR-26a-5p or RNU44 normalization on miR-335-3p expression in PC, DC, and HYP samples. Values are shown as delta-Ct with respect to normalizing reference genes (RGs). # stands for *p*-value = 0.1, * for *p*-value ≤ 0.05, ** for *p*-value < 0.01, *** for *p*-value < 0.001, and **** for *p*-value < 0.0001. (**C**) Pearson correlation scatter plots for miR-335-3p and miR-26a-5p/RNU44 RGs. x and y-axis indicate Ct values.

The effects of best/worst normalization strategies on target miRNA profiles were evaluated. The expression levels were computed using either the best (miR-26a-5p) or the worst (miR-212-3p) normalizers and compared with the results obtained with the normalization approach used in McAlinden analysis (RNU44) that also demonstrated its suitability in our ranking. Heat maps in Figure 3A clearly show that all three normalization approaches were able to cluster a group composed of H24243-HYP, H23689-PC, H23814-HYP, H23387-PC, and H23731-DC samples. The hierarchy in this group was maintained for miR-26a-5p and RNU44, while changed using miR-212-3p. Further, with the wrong normalization approach, an apparent and erroneous overexpression of almost all miRNAs in H24243-PC/DC samples emerged. Moreover, despite similar gross outcomes for the two most stable RGs, defined by the identification of those samples clearly different from the others, small rearrangement in both samples and miRNAs dendrograms could be observed, confirming that a most refined strategy allows identifying more specific co-regulation.

To evaluate the crucial impact of the best strategy on the reliable quantification of subtle discrepancies between cartilage regions, a more refined analysis was performed on miR-335-3p levels. miR-335-3p was selected since in McAlinden analysis, performed using RNU44, it was reported as significantly modulated within PC, DC, and HYP samples. In Figure 3B, it clearly appears that in a context of similar outcomes between the two approaches, the use of the most reliable miR-26a-5p does not allow scoring a significant (*p*-value ≤ 0.05) difference between PC and DC samples. To rule out this variation, correlation analysis for miR-26a-5p, RNU44, and miR-335-3p was performed across all samples. Interestingly, RNU44 and miR-335-3p showed a higher correlation coefficient (R^2^ = 0.68 vs. 0.53 for miR-26a-5p/miR-335-3p) (Figure 3C), leading to more homogenous ratios with reduced standard deviation and eventually higher statistical significance. Notably, the correlation between miR-26a-5p and RNU44 was high (0.82) as expected for RGs with similar stability profiles, again emphasizing that from small differences, major distortions may arise.

## 3. Discussion

The present work identified miR-26a-5p as a suitable RG for studies on miRNAs involved in and controlling the developmental process of human cartilage. By a multi-technique quantitative approach, we also demonstrated the good performance of the widely-used normalizer RNU44, in this specific experimental context. The application of different normalization strategies in the exemplary case of miR-335-3p assessment pointed out the criticality of the RG choice to obtain significant information on miRNA modulation in cartilage development.

Normalization strategy is an open question in studies assessing miRNA stability and comparison between samples from different sources or donors. Traditionally, in qRT-PCR studies, the relative quantification method is used, comparing expression levels of target miRNAs with the amount of an endogenous RG. In this context, small nuclear/nucleolar RNAs (snRNAs, e.g., RNU6, RNU44, or RNU48) have been commonly preferred [20]. This approach has some advantages, as well as important pitfalls. In fact, small RNA molecules, such as miRNAs and snRNAs, share similar features, such as stability and size. Further, snRNAs are ubiquitous and abundantly expressed, crucial traits for a reliable RG [21]. Nevertheless, snRNA biogenesis is mechanistically separated from miRNA biogenesis. As an example, RNU6 is not processed by the spliceosome but by the Drosha complex and does not mirror the physicochemical properties of miRNA molecules [22]. Therefore, the selection of the reference RNA on the class of RNAs being investigated is a fundamental issue, suggesting that reference miRNAs would be preferred for miRNAs.

To date, no universal miRNA RGs have been proposed due to high variability between donors, tissues, and developmental stages, making miRNA studies results largely incomparable [23]. Therefore, the selection of RGs that are reliable within the experimental condition under analysis is a pivotal pre-requisite. In this perspective, for large miRNA datasets, the global mean expression value normalization was proposed as highly effective, in terms of both reduction of technical variation and more accurate quantification of biological fluctuations [24]. However, when only a limited number of miRNA molecules are profiled, the selection of specific RGs is mandatory. To answer this need, the identification of invariant miRNAs by algorithms, specifically developed for RG evaluation and selection, resulted in a promising strategy [16,17,18,19].

In the field of developmental biology, such strategy has been successfully applied in a few studies in the plant or animal systems [25,26,27,28,29]. In human studies, a major issue is an availability of starting tissue material, especially when developmental studies are performed at embryonic stages. In the present work, the valuable data regarding miRNA expression in embryonic cartilage fragments, containing either precursor, differentiated, or hypertrophic chondrocytes [15] was mined. RNU44, used by McAlinden group as a normalizer for differentially-expressed miRNAs, was a reliable RG, ranking second when all samples were grouped, and always within the first ten positions in the separated cartilage regions. From our analysis, miR-26a-5p emerged as the most accurate RG, standing first for the analysis of all samples together, and in the top five for disjointed isolates. At present, no reports connecting miR-26a-5p and cartilage development are known, reinforcing its suitability as stable RG, although more focused studies are needed. In a wider context related to cartilage, in human chondrocytes, the downregulation of miR-26a-5p expression by IL-1b through nuclear factor kappa B (NF-kB) enhanced the production of OA-related nitric oxide synthase 2 (iNOS) protein, due to the ability of miR-26a-5p to directly target iNOS mRNA 3’UTR [30]. Therefore, although stable in developing cartilage, miR-26a-5p reliability as chondrocyte miRNA RG in adult tissues under inflammation or OA might be considered carefully, and our suggestion is to verify, through the herein proposed algorithms, a panel of miRNA RGs, if possible selected from studies in the same or similar field.

The marked differences in performance of the tested normalization strategies (miR-26a-5p/RNU44/miR-212-3p) had a significant impact on miRNAs profiling in the different regions of developing cartilage, possibly precluding accurate biological implications. Although all normalization strategies allowed to clearly distinguish a subgroup of markedly different samples, either best or worst RGs completely changed the arrangement of both sample and miRNA dendrograms (Figure 3A). Moreover, even using miR-26a-5p, it was not possible to clearly cluster PC, DC, and HYP regions. This may indicate that overall, or at least for the 46 scored miRNAs, no fingerprinting major transcriptional differences are present. In this context of global high similarity, few miRNAs may be crucial to support biological outcomes, and reliable and refined evaluation of small variations gets decisive, as shown for miR-335-3p, in assessing significant modulations.

In conclusion, this study pointed out for the first time the reliability of miR-26a-5p for future studies aimed at dissecting new or low abundant miRNAs regulation in different regions of human developing cartilage. The main limitation of the report is the lack of validation of the proposed analysis on independent samples. Therefore, future evaluation of miRNA expression from developing cartilage should be performed on the path of herein proposed candidates, possibly from multiple samples for each individual to reduce overall variability [31].

## 4. Materials and Methods 

### 4.1. Data Retrieval and Ethics Statement

qRT-PCR data can be found in the GEO database [32], with the record GSE49152 [15]. Only miRNAs with reported Ct values in all samples were considered for the analysis (Appendix A Table A1, Table A2 and Table A3). For the donors, briefly, human, normal embryonic tissue samples (limbs) at gestational day 54–56 were obtained from nine donors, and laser capture microdissection was performed to obtain tissue from the precursor chondrocyte (PC), differentiated chondrocyte (DC), or hypertrophic chondrocyte (HYP) regions. After RNA extraction, TaqMan^®^ OpenArray^®^ technology was used to determine microRNA expression profiles starting from 30 ng total RNA for each sample (Thermo Fisher Scientific, Waltham, MA, USA). TaqMan^®^ OpenArray^®^ technology is a fixed-content panel containing 754 validated human TaqMan^®^ MicroRNA Assays derived from Sanger miRBase release v.14. The panel is specifically designed to provide specificity for only the mature miRNA targets. TaqMan MicroRNA Assays incorporate a target-specific stem-loop reverse transcription primer allowing to work despite the short length of mature miRNAs (~22 nucleotides).

As stated in McAlinden work, the Human Research Protection Office (HRPO) at Washington University in St Louis reviewed the request to work with human embryonic tissue, and the original project was deemed exempt since it did not constitute human subjects research and receiving embryonic tissue from the University of Washington would not involve obtaining data through intervention or interaction with a living individual, and, other than gestational age, no identifying information was provided upon receipt of the tissue.

### 4.2. Assessment of RG Stability

Gene expression stability was evaluated according to four gold-standard statistical approaches: BestKeeper [16], geNorm [17], NormFinder [18], and the comparative delta-Ct method [19]. BestKeeper analysis uses Ct values directly, while geNorm, NormFinder, and delta-Ct method use transformed Ct values of (1 + E) − ΔCt. The ranking of the RGs according to their stability was generated by each algorithm, and a series of continuous integers starting from 1 was assigned to each RG. The overall performance of the miRNA RGs was evaluated by combining the results of the four approaches through a global ranking obtained as the geometric mean of the rankings given by each analysis [33,34].

### 4.3. Statistical Analyses

Statistical analyses were performed using GraphPad Prism Software version 5 (GraphPad, San Diego, CA, USA). Presence of outliers was scored by Grubbs’ test. When two sets of data (PCvsDC, PCvsHYP, DCvsHYP) were compared, as in McAlinden paper, the comparison was performed by using unpaired Student t-test. Significance level was set at *p*-value ≤ 0.05. Pearson correlation coefficient (R^2^) was estimated to determine the linear association between samples or miRNA Ct values. The outcome results were interpreted according to the degree of association [35].

Heatmaps were generated scoring Cycle relative threshold (Crt) values normalized both with stable miR-26a-5p/RNU44 and unstable miR-212-3pwith ClustVis package (https://biit.cs.ut.ee/clustvis/) [36]. After row centering, maps were generated using the following settings for both rows and columns clustering distance and method: correlation and average, respectively.

## Abbreviations

qRT-PCR	Quantitative Real-Time Polymerase Chain Reaction
RG	Reference Gene
OA	Osteoarthritis
miRNA	microRNA
PC	precursor chondrocytes
DC	differentiated chondrocytes
HYP	hypertrophic chondrocytes
VEGF	vascular endothelial growth factor
IGF-1	insulin-like growth factor-1
TGF-β	transforming growth factor beta
BMP	bone morphogenetic protein
FGF	fibroblast growth factor

## Appendix A

**Table A1:** Distribution of the quantification cycles (Crt) values across precursor chondrocytes (PC) samples.

ID_REF	H23374	H23387	H23689	H23731	H23804	H24010	H24051	H24243	Mean	SD
miR-16-5p	15.76	17.263	14.612	17.995	15.589	16.245	16.118	15.243	16.103	1.088
miR-19a-3p	17.769	12.165	8.759	18.885	16.404	17.197	17.497	16.911	15.698	3.434
miR-25-3p	16.781	13.398	10.264	17.825	16.354	16.019	16.226	14.745	15.202	2.398
miR-26a-5p	13.795	14.585	10.863	14.655	12.569	12.826	12.921	12.091	13.038	1.281
miR-26b-5p	15.127	15.954	11.639	15.812	13.683	13.633	14.025	14.717	14.324	1.407
miR-30d-5p	18.15	18.348	18.033	18.384	16.167	15.424	15.907	16.049	17.058	1.275
miR-99b-5p	13.355	14.608	11.317	14.06	12.283	12.488	12.877	32.047	15.379	6.813
miR-190a	22.433	19.043	16.729	22.04	21.151	20.133	20.519	20.133	20.273	1.802
miR-192-5p	19.521	20.214	17.789	21.362	18.292	19.292	19.359	30.646	20.809	4.122
miR-211-5p	16.856	14.045	15.184	16.354	17.323	16.399	16.815	16.893	16.234	1.088
miR-212-3p	15.3	25.443	21.018	14.492	15.321	15.068	15.161	32.376	19.272	6.565
miR-221-3p	21.038	16.549	12.994	23.498	21.289	19.233	20.512	18.489	19.200	3.251
miR-296-5p	17.126	16.506	12.682	16.368	15.281	15.512	15.894	13.817	15.398	1.479
miR-301a-3p	17.437	24.183	20.688	17.512	15.668	15.564	15.632	15.073	17.720	3.183
miR-328	16.275	20.79	17.911	16.196	15.318	15.291	15.35	16.314	16.681	1.871
miR-331-3p	14.993	14.602	11.136	15.003	13.6	13.86	14.027	12.325	13.693	1.352
miR-369-3p	22.2	25.225	19.24	22.316	20.755	19.644	20.457	21.571	21.426	1.897
miR-373-3p	20.872	17.352	13.565	18.928	20.73	18.879	19.095	16.799	18.278	2.374
miR-365	18.866	26.069	22.047	20.417	18.557	18.724	18.712	20.156	20.444	2.572
miR-520b	22.805	24.426	24.831	22.453	24.983	21.584	21.652	22.462	23.150	1.393
miR-542-3p	21.685	18.276	15.802	22.499	20.563	19.7	20.214	20.529	19.909	2.082
miR-659-3p	24.314	15.082	11.816	22.937	24.641	19.723	20.466	22.921	20.238	4.603
miR-758	19.959	17.303	15.235	22.147	20.961	21.27	20.411	21.351	19.830	2.357
miR-769-5p	20.453	20.809	20.585	21.329	19.666	20.208	20.169	20.087	20.413	0.506
miR-362-3p	21.876	31.59	27.068	23.274	21.24	21.177	21.92	22.259	23.801	3.678
miR-339-3p	21.266	16.579	13.694	22.451	20.253	22.428	21.516	22.011	20.025	3.193
miR-335-3p	18.111	18.732	15.78	17.727	16.023	17.845	17.513	17.737	17.434	1.016
miR-345-5p	18.021	20.615	17.911	19.548	17.585	18.684	18.124	20.252	18.843	1.152
miR-886-5p	18.156	15.614	14.222	18.138	16.017	15.215	14.268	34.51	18.268	6.736
miR-323-3p	17.171	19.577	15.798	18.177	16.057	17.305	17.291	16.15	17.191	1.253
miR-151a-3p	19.206	15.52	14.021	21.469	18.197	19.953	19.181	20.01	18.445	2.484
miR-340-3p	21.096	16.028	14.812	22.218	20.309	21.181	21.625	20.294	19.695	2.733
miR-342-3p	16.005	18.656	16.827	17.393	14.8	17.462	16.833	15.172	16.644	1.272
miR-193a-5p	20.281	19.074	16.07	21.22	20.044	19.475	19.573	20.068	19.476	1.518
miR-138-5p	20.323	17.887	16.26	22.691	20.647	20.028	20.853	19.036	19.716	1.972
miR-199a-3p	12.892	24.971	21.137	14.097	11.852	11.869	12.326	11.159	15.038	5.126
miR-10b-3p	18.889	23.797	20.055	20.166	16.796	18.28	19.337	18.034	19.419	2.088
miR-483-3p	16.064	20.652	18.422	16.133	14.348	16.081	16.253	15.515	16.684	1.958
miR-202-3p	20.34	19.782	16.845	20.327	20.869	20.395	20.38	25.763	20.588	2.442
miR-494	18.156	24.034	20.525	19.472	18.219	19.466	18.362	21.292	19.941	2.000
miR-193b-5p	23.232	15.684	12.657	24.236	22.339	23.219	23.33	24.496	21.149	4.432
miR-543	18.858	21.579	19.426	19.625	17.319	18.876	18.825	17.496	19.001	1.330
miR-155-5p	18.446	21.61	17.761	18.895	18.45	18.053	18.717	15.747	18.460	1.612
miR-1291	20.252	17.87	16.122	20.902	20.273	19.908	18.814	20.51	19.331	1.631
miR-1275	17.891	23.592	23.873	18.257	17.67	17.119	16.898	17.023	19.040	2.933
miR-720	13.546	18.461	18.539	13.048	11.742	11.685	12.233	10.165	13.677	3.141
RNU44	14.806	14.132	12.033	15.060	12.878	14.185	14.610	14.081	13.973	1.023

**Table A2:** Distribution of the quantification cycles (Crt) values across differentiated chondrocytes (DC) samples.

ID_REF	H23374	H23387	H23689	H23731	H23804	H23814	H24010	H24051	H24243	Mean	SD
miR-16-5p	17.12	19.173	13.827	19.497	15.103	17.281	15.051	18.18	17.023	16.917	1.932
miR-19a-3p	19.173	19.646	14.969	14.039	15.501	18.006	16.431	19.486	18.396	17.294	2.108
miR-25-3p	18.26	19.566	14.254	15.571	15.876	18.407	15.604	18.32	17.383	17.027	1.762
miR-26a-5p	14.456	15.767	10.853	16.905	12.028	14.485	12.264	14.734	14.021	13.946	1.915
miR-26b-5p	16.067	17.028	11.617	18.668	13.406	16.199	13.601	16.473	16.741	15.533	2.200
miR-30d-5p	18.621	19.887	14.616	20.917	16.24	18.469	15.931	18.025	18.159	17.874	1.981
miR-99b-5p	14.476	15.482	10.879	17.203	11.883	14.102	12.509	14.976	19.153	14.518	2.604
miR-190a	23.051	22.927	16.937	22.253	21.262	22.594	19.881	21.78	22.958	21.516	1.997
miR-192-5p	21.866	21.433	17.511	22.669	18.409	20.227	18.215	21.526	23.971	20.647	2.206
miR-211-5p	17.132	16.976	14.709	14.849	16.078	17.864	16.124	17.551	18.711	16.666	1.346
miR-212-3p	14.613	13.779	12.999	24.595	13.138	14.085	13.629	16.116	23.3	16.250	4.472
miR-221-3p	20.669	23.643	15.289	17.863	18.2	21.544	17.989	22.264	20.454	19.768	2.622
miR-296-5p	18.069	18.4	14.146	18.503	14.95	16.135	15.305	17.273	17.175	16.662	1.597
miR-301a-3p	18.064	18.517	13.829	23.646	14.813	17.132	14.529	17.745	17.083	17.262	2.926
miR-328	16.475	17.018	14.19	22.472	14.142	15.911	14.684	16.332	17.017	16.471	2.519
miR-331-3p	15.569	16.34	12.158	17.457	12.659	14.506	13.256	15.689	15.093	14.747	1.765
miR-369-3p	22.614	22.705	17.327	25.569	19.288	21.306	18.575	21.924	22.402	21.301	2.520
miR-373-3p	20.674	19.904	16.998	19.633	19.27	21.31	17.897	19.903	20.208	19.533	1.340
miR-365	18.78	19.767	15.079	28.769	16.344	17.797	16.243	18.518	18.977	18.919	3.996
miR-520b	22.799	21.624	19.716	33.414	22.364	23.634	22.654	21.946	20.935	23.232	3.984
miR-542-3p	21.582	23.098	17.779	20.597	19.413	21.347	18.568	21.172	21.577	20.570	1.678
miR-659-3p	23.839	25.276	25.615	18.596	24.974	24.452	20.877	22.417	24.389	23.382	2.334
miR-758	21.995	23.84	18.765	18.623	19.302	20.851	19.586	22.638	22.458	20.895	1.914
miR-769-5p	21.339	22.454	18.869	22.063	19.044	21.069	19.638	22.079	22.089	20.960	1.411
miR-362-3p	23.264	25.611	19.183	30.957	21.065	24.316	21.961	24.489	27.286	24.237	3.503
miR-339-3p	22.631	25.648	19.203	20.886	19.583	21.204	20.602	23.122	23.487	21.818	2.073
miR-335-3p	20.206	20.566	16.702	19.991	16.541	18.369	18.316	21.196	20.954	19.205	1.780
miR-345-5p	19.194	20.928	16.144	23.486	16.185	18.263	17.547	19.926	20.206	19.098	2.367
miR-886-5p	17.998	19.349	15.829	16.404	14.59	17.109	13.063	15.461	20.095	16.655	2.248
miR-323-3p	18.193	21.046	16.7	20.679	15.28	18.114	16.436	20.365	19.267	18.453	2.043
miR-151a-3p	21.498	22.716	17.214	18.35	17.951	20.003	18.789	21.554	21.878	19.995	1.991
miR-340-3p	22.248	23.312	18.799	18.859	19.4	21.991	20.369	23.269	21.824	21.119	1.802
miR-342-3p	17.528	19.571	14.822	30.514	14.624	16.711	16.143	18.977	17.413	18.478	4.811
miR-193a-5p	20.548	23.09	18.877	22.735	19.239	21.038	18.522	20.77	21.864	20.743	1.641
miR-138-5p	17.679	20.05	14.523	21.741	15.573	17.727	14.985	17.7	17.133	17.457	2.335
miR-199a-3p	14.285	15.795	10.526	28.462	11.458	14.423	12.243	14.979	14.41	15.176	5.279
miR-10b-3p	21.266	22.542	16.191	26.342	18.356	20.841	18.43	22.372	21.266	20.845	2.947
miR-483-3p	17.108	18.086	15.868	25.096	15.009	15.966	16.949	18.263	17.59	17.771	2.951
miR-202-3p	20.522	20.408	19.698	20.947	19.797	20.571	19.366	20.643	22.89	20.538	1.022
miR-494	18.3	22.234	16.351	24.418	17.997	18.644	17.753	19.537	20.287	19.502	2.492
miR-193b-5p	22.426	24.428	19.749	14.727	21.926	22.503	20.477	22.748	23.226	21.357	2.849
miR-543	19.725	20.497	16.45	24.05	16.957	19.71	18.215	20.644	19.111	19.484	2.255
miR-155-5p	19.397	19.848	16.905	23.729	17.946	19.621	18.419	19.466	18.209	19.282	1.923
miR-1291	20.157	21.16	18.004	18.882	18.388	20.321	17.279	18.851	19.992	19.226	1.257
miR-1275	18.413	19.438	17.212	23.706	17.532	17.948	16.416	18.798	17.974	18.604	2.107
miR-720	14.054	14.578	10.717	19.77	11.537	12.671	11.917	13.545	11.266	13.339	2.746
RNU44	15.035	15.900	11.991	16.028	12.189	14.030	13.579	15.677	15.308	14.415	1.552

**Table A3:** Distribution of the quantification cycles (Crt) values across hypertrohic chondrocytes (HYP) samples.

ID_REF	H23374	H23387	H23689	H23731	H23804	H23814	H24010	H24051	H24243	Mean	SD
miR-16-5p	18.528	28.879	16.163	21.854	16.735	17.016	17.585	18.541	20.45	19.528	3.951
miR-19a-3p	18.582	21.88	15.871	21.321	16.335	11.963	17.048	18.054	14.71	17.307	3.114
miR-25-3p	18.541	22.189	15.481	21.375	18.069	14.712	17.537	18.427	16.241	18.064	2.496
miR-26a-5p	14.922	18.867	11.822	17.5	13.602	16.324	13.73	14.296	19.241	15.589	2.549
miR-26b-5p	16.391	19.673	12.735	19.524	15.148	17.175	15.39	16.327	19.157	16.836	2.318
miR-30d-5p	18.549	23.304	15.538	21.844	18.124	18.559	17.22	17.752	22.519	19.268	2.651
miR-99b-5p	15.05	18.32	12.083	17.595	13.425	16.828	14.05	15.091	22.232	16.075	3.060
miR-190a	22.098	25.533	18.38	23.351	22.828	18.971	20.521	21.379	29.678	22.527	3.470
miR-192-5p	20.601	24.885	17.834	24.027	18.671	21.408	19.425	19.713	23.936	21.167	2.563
miR-211-5p	16.568	17.604	15.181	17.976	16.704	15.435	16.419	17.368	22.462	17.302	2.146
miR-212-3p	14.257	14.947	12.639	16.028	14.471	20.82	13.94	14.609	22.886	16.066	3.438
miR-221-3p	19.8	30.603	16.157	23.446	18.198	17.511	18.256	19.528	18.71	20.245	4.372
miR-296-5p	19.405	21.089	15.01	19.641	16.887	17.988	16.437	17.111	20.606	18.242	2.058
miR-301a-3p	19.532	23.071	15.956	21.837	17.489	27.545	17.101	18.399	23.971	20.545	3.819
miR-328	16.474	19.028	14.579	18.042	15.126	21.652	15.36	15.923	22.1	17.587	2.812
miR-331-3p	16.127	18.497	12.725	17.333	13.622	18.226	14.236	15.254	21.06	16.342	2.683
miR-369-3p	21.945	27.602	18.699	25.399	21.518	23.821	20.73	21.763	19.457	22.326	2.841
miR-373-3p	19.909	21.263	17.472	21.982	20.496	17.689	18.828	19.719	19.944	19.700	1.506
miR-365	19.141	24.593	15.782	20.998	17.907	26.623	16.523	17.53	30.747	21.094	5.145
miR-520b	22.05	21.389	20.184	23.525	22.721	33.405	21.016	21.654	12.988	22.104	5.230
miR-542-3p	22.391	27.024	19.535	24.99	21.464	18.687	20.502	21.687	22.607	22.099	2.605
miR-659-3p	22.515	30.9	23.112	26.067	25.795	18.229	21.82	22.5	22.353	23.699	3.541
miR-758	27.024	33.204	20.866	27.259	21.531	17.753	21.544	23.32	20.466	23.663	4.709
miR-769-5p	22.031	27.52	19.756	24.831	20.807	21.286	20.802	21.854	24.272	22.573	2.478
miR-362-3p	24.469	27.73	21.842	27.93	23.975	30.514	23.431	24.455	28.147	25.833	2.832
miR-339-3p	22.62	28.911	20.67	25.379	21.062	22.864	21.405	22.889	25.914	23.524	2.705
miR-335-3p	23.272	29.163	19.571	26.107	21.129	18.335	23.277	23.334	22.887	23.008	3.258
miR-345-5p	19.747	24.72	17.445	22.648	18.367	21.434	18.755	19.531	27.505	21.128	3.302
miR-886-5p	16.516	20.772	13.932	17.941	14.419	13.708	12.258	13.314	15.626	15.387	2.665
miR-323-3p	19.445	26.756	17.451	24.573	17.575	14.899	19.091	19.708	21.803	20.145	3.695
miR-151a-3p	22.117	27.1	19.211	24.539	19.054	15.677	19.737	20.356	18.396	20.687	3.432
miR-340-3p	22.684	29.818	20.16	25.137	21.236	16.251	22.248	23.47	19.421	22.269	3.819
miR-342-3p	18.531	22.53	15.9	20.756	15.486	29.993	18.261	18.011	32.656	21.347	6.093
miR-193a-5p	20.491	24.301	18.297	22.426	19.566	21.331	18.856	19.258	25.898	21.158	2.599
miR-138-5p	17.91	21.729	15.212	19.901	16.63	17.693	15.111	16.293	34.84	19.480	6.148
miR-199a-3p	14.901	20.186	11.839	17.593	12.582	25.568	13.809	14.883	31	18.040	6.475
miR-10b-3p	21.556	24.427	18.741	24.167	18.979	23.849	20.676	21.789	27.299	22.387	2.795
miR-483-3p	20.301	22.767	19.374	21.706	18.339	23.61	19.891	20.168	31.851	22.001	4.051
miR-202-3p	19.543	22.099	18.483	21.209	19.729	20.273	19.692	20.053	23.314	20.488	1.477
miR-494	19.363	30.249	18.016	26.464	19.706	22.744	19.094	19.281	23.698	22.068	4.112
miR-193b-5p	23.994	27.276	19.598	24.023	22.118	12.469	20.526	21.713	15.242	20.773	4.568
miR-543	20.536	23.678	18.092	23.71	19.499	24.266	20.128	20.697	26.157	21.863	2.666
miR-155-5p	19.587	18.328	17.54	19.649	17.296	23.726	19.085	19.539	25.65	20.044	2.813
miR-1291	18.728	22.494	17.419	21.682	18.98	19.234	17.506	17.481	19.804	19.259	1.824
miR-1275	18.351	22.249	17.141	20.426	18.859	24.48	17.453	18.348	26.251	20.395	3.248
miR-720	13.064	15.841	11.024	14.008	11.498	17.967	12.458	12.451	20.048	14.262	3.079
RNU44	15.843	18.931	12.639	17.203	13.430	14.392	14.658	15.450	17.585	15.570	2.046

**Table A4:** miRNA stability ranking in precursor chondrocytes (PC).

Ranking	Geomean		BestKeeper		geNorm		delta-Ct		NormFinder	
1	RNU44	2.63	miR-769-5p	0.38	miR-296-5p | miR-331-3p	0.25	RNU44	2.4	miR-26b-5p	0.452
2	miR-335-3p	3.6	RNU44	0.76			miR-26a-5p	2.4	miR-335-3p	0.516
3	miR-26b-5p	3.81	miR-335-3p	0.77	miR-26a-5p	0.456	miR-26b-5p	2.41	miR-345-5p	0.594
4	miR-26a-5p	4.26	miR-16-5p	0.8	miR-16-5p	0.629	miR-335-3p	2.42	RNU44	0.597
5	miR-16-5p	4.68	miR-211-5p	0.81	miR-26b-5p	0.711	miR-16-5p	2.42	miR-26a-5p	0.785
6	miR-769-5p	4.88	miR-323-3p	0.9	RNU44	0.755	miR-323-3p	2.47	miR-16-5p	0.798
7	miR-331-3p	5.49	miR-543	0.91	miR-335-3p	0.789	miR-331-3p	2.47	miR-769-5p	0.832
8	miR-296-5p	6.53	miR-193a-5p	0.95	miR-323-3p	0.809	miR-193a-5p	2.48	miR-193a-5p	0.96
9	miR-323-3p	7.14	miR-155-5p	0.96	miR-769-5p	0.875	miR-769-5p	2.48	miR-323-3p	0.97
10	miR-345-5p	7.75	miR-345-5p	0.97	miR-342-3p	0.954	miR-345-5p	2.52	miR-331-3p	1.055
11	miR-193a-5p	9.51	miR-26a-5p	0.98	miR-543	1.003	miR-296-5p	2.55	miR-296-5p	1.217
12	miR-543	11.25	miR-342-3p	0.99	miR-345-5p	1.043	miR-1291	2.62	miR-369-3p	1.255
13	miR-211-5p	12.83	miR-331-3p	1	miR-155-5p	1.092	miR-543	2.66	miR-1291	1.272
14	miR-342-3p	13.23	miR-26b-5p	1.08	miR-30d-5p	1.14	miR-190a	2.66	miR-30d-5p	1.41
15	miR-155-5p	14.71	miR-296-5p	1.1	miR-369-3p	1.184	miR-342-3p	2.69	miR-211-5p	1.426
16	miR-30d-5p	15.2	miR-30d-5p	1.17	miR-193a-5p	1.23	miR-369-3p	2.72	miR-543	1.458
17	miR-1291	15.2	miR-520b	1.2	miR-190a	1.295	miR-30d-5p	2.72	miR-342-3p	1.462
18	miR-369-3p	15.87	miR-190a	1.26	miR-1291	1.347	miR-542-3p	2.74	miR-190a	1.487
19	miR-190a	16.66	miR-1291	1.3	miR-211-5p	1.397	miR-211-5p	2.78	miR-542-3p	1.647
20	miR-542-3p	20.34	miR-328	1.33	miR-542-3p	1.443	miR-155-5p	2.8	miR-155-5p	1.712
21	miR-138-5p	22.15	miR-202-3p	1.36	miR-138-5p	1.477	miR-138-5p	2.81	miR-138-5p	1.792
22	miR-328	22.2	miR-369-3p	1.4	miR-10b-3p	1.537	miR-328	2.94	miR-494	1.87
23	miR-520b	23.07	miR-483-3p	1.43	miR-328	1.588	miR-25-3p	2.95	miR-520b	1.908
24	miR-10b-3p	23.96	miR-10b-3p	1.44	miR-483-3p	1.634	miR-10b-3p	3	miR-328	1.914
25	miR-494	25.17	miR-542-3p	1.49	miR-520b	1.675	miR-758	3	miR-202-3p	1.957
26	miR-483-3p	26.09	miR-138-5p	1.49	miR-494	1.715	miR-494	3.03	miR-10b-3p	2.034
27	miR-202-3p	26.86	miR-494	1.51	miR-25-3p	1.761	miR-151a-3p	3.06	miR-758	2.059
28	miR-25-3p	26.87	miR-373-3p	1.78	miR-373-3p	1.805	miR-483-3p	3.06	miR-25-3p	2.106
29	miR-758	27.45	miR-758	1.78	miR-758	1.847	miR-520b	3.06	miR-151a-3p	2.153
30	miR-373-3p	29.22	miR-25-3p	1.8	miR-151a-3p	1.887	miR-373-3p	3.14	miR-483-3p	2.154
31	miR-151a-3p	29.44	miR-365	1.81	miR-340-3p	1.934	miR-202-3p	3.23	miR-373-3p	2.258
32	miR-340-3p	32.24	miR-151a-3p	1.9	miR-202-3p	1.983	miR-340-3p	3.28	miR-365	2.544
33	miR-365	32.95	miR-340-3p	2.14	miR-221-3p	2.037	miR-365	3.35	miR-340-3p	2.567
34	miR-221-3p	34.73	miR-1275	2.35	miR-339-3p	2.086	miR-339-3p	3.54	miR-339-3p	2.878
35	miR-339-3p	34.96	miR-301a-3p	2.36	miR-19a-3p	2.134	miR-221-3p	3.58	miR-221-3p	2.952
36	miR-301a-3p	36.49	miR-221-3p	2.39	miR-365	2.186	miR-19a-3p	3.67	miR-19a-3p	3.098
37	miR-19a-3p	36.7	miR-720	2.41	miR-301a-3p	2.262	miR-301a-3p	3.88	miR-301a-3p	3.339
38	miR-1275	36.96	miR-339-3p	2.44	miR-1275	2.332	miR-1275	3.92	miR-1275	3.346
39	miR-720	38.49	miR-192-5p	2.6	miR-720	2.399	miR-720	4.08	miR-720	3.574
40	miR-362-3p	40.5	miR-19a-3p	2.62	miR-362-3p	2.472	miR-362-3p	4.26	miR-192-5p	3.659
41	miR-192-5p	40.72	miR-362-3p	2.76	miR-193b-5p	2.562	miR-192-5p	4.5	miR-362-3p	3.775
42	miR-193b-5p	41.75	miR-193b-5p	3.49	miR-659-3p	2.652	miR-193b-5p	4.57	miR-193b-5p	4.137
43	miR-659-3p	42.75	miR-659-3p	3.52	miR-192-5p	2.739	miR-659-3p	4.79	miR-659-3p	4.331
44	miR-199a-3p	44	miR-199a-3p	4.01	miR-199a-3p	2.862	miR-199a-3p	5.67	miR-199a-3p	5.386
45	miR-886-5p	45	miR-886-5p	4.06	miR-886-5p	3.044	miR-886-5p	6.78	miR-886-5p	6.388
46	miR-212-3p	46.25	miR-99b-5p	4.17	miR-212-3p	3.212	miR-212-3p	6.85	miR-212-3p	6.407
47	miR-99b-5p	46.75	miR-212-3p	5.25	miR-99b-5p	3.367	miR-99b-5p	6.85	miR-99b-5p	6.463

**Table A5:** miRNA stability ranking in differentiated chondrocytes (DC).

Ranking	Geomean		BestKeeper		geNorm		delta-Ct		NormFinder	
1	miR-331-3p	2.63	miR-202-3p	0.64	miR-16-5p | miR-26a-5p	0.302	miR-331-3p	1.61	miR-331-3p	0.179
2	miR-26a-5p	2.74	miR-373-3p	0.99			miR-26a-5p	1.63	miR-26a-5p	0.309
3	miR-16-5p	3.66	miR-1291	1.05	miR-30d-5p	0.366	miR-16-5p	1.65	miR-30d-5p	0.398
4	miR-30d-5p	4.97	miR-211-5p	1.09	miR-331-3p	0.421	miR-30d-5p	1.66	miR-16-5p	0.414
5	miR-296-5p	6.51	miR-769-5p	1.18	miR-26b-5p	0.493	miR-296-5p	1.67	miR-26b-5p	0.474
6	RNU44	6.96	miR-155-5p	1.26	miR-296-5p	0.564	RNU44	1.68	miR-296-5p	0.492
7	miR-769-5p	7.09	miR-193a-5p	1.29	RNU44	0.614	miR-769-5p	1.68	RNU44	0.542
8	miR-193a-5p	8.21	RNU44	1.3	miR-769-5p	0.652	miR-26b-5p	1.69	miR-193a-5p	0.574
9	miR-26b-5p	8.65	miR-542-3p	1.32	miR-193a-5p	0.692	miR-193a-5p	1.72	miR-769-5p	0.604
10	miR-202-3p	11.73	miR-296-5p	1.36	miR-138-5p	0.75	miR-345-5p	1.79	miR-345-5p	0.776
11	miR-373-3p	13.03	miR-1275	1.36	miR-345-5p	0.798	miR-138-5p	1.82	miR-369-3p	0.832
12	miR-155-5p	13.07	miR-331-3p	1.42	miR-543	0.832	miR-369-3p	1.83	miR-138-5p	0.848
13	miR-138-5p	13.34	miR-190a	1.44	miR-369-3p	0.858	miR-543	1.83	miR-323-3p	0.871
14	miR-345-5p	13.7	miR-26a-5p	1.49	miR-323-3p	0.886	miR-542-3p	1.85	miR-543	0.89
15	miR-1291	14.39	miR-16-5p	1.5	miR-155-5p	0.928	miR-323-3p	1.85	miR-192-5p	1.013
16	miR-543	14.63	miR-25-3p	1.51	miR-494	0.968	miR-335-3p	1.88	miR-335-3p	1.029
17	miR-542-3p	14.9	miR-30d-5p	1.52	miR-10b-3p	1.006	miR-192-5p	1.93	miR-542-3p	1.078
18	miR-369-3p	15.77	miR-335-3p	1.53	miR-1275	1.04	miR-155-5p	1.96	miR-155-5p	1.086
19	miR-323-3p	15.83	miR-340-3p	1.57	miR-328	1.069	miR-494	1.99	miR-494	1.141
20	miR-1275	16.98	miR-328	1.58	miR-301a-3p	1.095	miR-190a	2.02	miR-1275	1.243
21	miR-335-3p	17.64	miR-543	1.6	miR-335-3p	1.13	miR-1275	2.04	miR-190a	1.245
22	miR-190a	19.03	miR-758	1.63	miR-192-5p	1.161	miR-1291	2.04	miR-10b-3p	1.301
23	miR-211-5p	20.06	miR-323-3p	1.68	miR-542-3p	1.192	miR-328	2.05	miR-328	1.31
24	miR-192-5p	20.25	miR-339-3p	1.69	miR-190a	1.228	miR-373-3p	2.05	miR-373-3p	1.335
25	miR-494	21.05	miR-138-5p	1.69	miR-373-3p	1.265	miR-10b-3p	2.06	miR-1291	1.376
26	miR-328	21.17	miR-151a-3p	1.71	miR-1291	1.298	miR-301a-3p	2.13	miR-202-3p	1.399
27	miR-10b-3p	24.73	miR-886-5p	1.76	miR-202-3p	1.331	miR-202-3p	2.13	miR-99b-5p	1.422
28	miR-25-3p	25.42	miR-26b-5p	1.77	miR-99b-5p	1.362	miR-339-3p	2.17	miR-301a-3p	1.466
29	miR-301a-3p	27.27	miR-483-3p	1.81	miR-339-3p	1.396	miR-25-3p	2.17	miR-339-3p	1.607
30	miR-339-3p	27.7	miR-19a-3p	1.83	miR-25-3p	1.426	miR-99b-5p	2.2	miR-25-3p	1.669
31	miR-340-3p	29.6	miR-192-5p	1.83	miR-151a-3p	1.454	miR-151a-3p	2.2	miR-151a-3p	1.726
32	miR-151a-3p	29.67	miR-659-3p	1.83	miR-758	1.487	miR-758	2.34	miR-720	1.75
33	miR-758	29.8	miR-345-5p	1.83	miR-340-3p	1.515	miR-720	2.34	miR-886-5p	1.884
34	miR-99b-5p	30.27	miR-494	1.88	miR-211-5p	1.541	miR-340-3p	2.35	miR-211-5p	1.893
35	miR-886-5p	32.55	miR-720	1.91	miR-886-5p	1.57	miR-211-5p	2.37	miR-758	1.934
36	miR-720	34.43	miR-99b-5p	1.94	miR-221-3p	1.602	miR-886-5p	2.46	miR-340-3p	1.945
37	miR-483-3p	35.74	miR-369-3p	1.94	miR-19a-3p	1.632	miR-483-3p	2.52	miR-362-3p	1.973
38	miR-19a-3p	36.81	miR-301a-3p	1.98	miR-720	1.663	miR-362-3p	2.54	miR-483-3p	2.002
39	miR-221-3p	38.71	miR-193b-5p	2.03	miR-362-3p	1.702	miR-221-3p	2.58	miR-221-3p	2.162
40	miR-362-3p	39.41	miR-10b-3p	2.12	miR-483-3p	1.739	miR-19a-3p	2.63	miR-19a-3p	2.322
41	miR-659-3p	40.48	miR-221-3p	2.16	miR-365	1.802	miR-365	3.05	miR-365	2.738
42	miR-365	41.49	miR-520b	2.35	miR-193b-5p	1.882	miR-520b	3.58	miR-520b	3.301
43	miR-193b-5p	42.19	miR-365	2.39	miR-659-3p	1.958	miR-193b-5p	3.75	miR-659-3p	3.54
44	miR-520b	42.49	miR-362-3p	2.55	miR-520b	2.035	miR-659-3p	3.77	miR-212-3p	3.565
45	miR-342-3p	45.25	miR-342-3p	3.03	miR-342-3p	2.118	miR-342-3p	3.82	miR-193b-5p	3.603
46	miR-212-3p	45.74	miR-199a-3p	3.09	miR-212-3p	2.196	miR-212-3p	3.9	miR-342-3p	3.64
47	miR-199a-3p	46.75	miR-212-3p	3.42	miR-199a-3p	2.282	miR-199a-3p	4.22	miR-199a-3p	4.076

**Table A6:** miRNA stability ranking in hypertrophic chondrocytes (HYP).

Ranking	Geomean		BestKeeper		geNorm		delta-Ct		NormFinder	
1	miR-26a-5p	2.89	miR-373-3p	1.14	miR-331-3p | miR-10b-3p	0.512	miR-26a-5p	2.33	miR-26a-5p	0.274
2	miR-192-5p	4.68	miR-202-3p	1.15			miR-192-5p	2.36	miR-192-5p	0.28
3	miR-26b-5p	5.63	miR-211-5p	1.38	miR-543	0.573	miR-30d-5p	2.38	miR-30d-5p	0.414
4	miR-30d-5p	6.43	miR-1291	1.38	miR-99b-5p	0.669	miR-26b-5p	2.4	miR-26b-5p	0.512
5	miR-331-3p	6.72	RNU44	1.62	miR-26a-5p	0.76	miR-769-5p	2.42	miR-296-5p	0.634
6	miR-296-5p	6.93	miR-296-5p	1.73	miR-193a-5p	0.818	miR-193a-5p	2.43	miR-339-3p	0.668
7	miR-10b-3p	7.57	miR-26b-5p	1.82	miR-345-5p	0.863	miR-296-5p	2.44	RNU44	0.708
8	RNU44	7.61	miR-25-3p	1.84	miR-192-5p	0.94	RNU44	2.44	miR-769-5p	0.722
9	miR-202-3p	7.9	miR-542-3p	1.92	miR-26b-5p	0.996	miR-339-3p	2.45	miR-193a-5p	0.73
10	miR-193a-5p	8.06	miR-769-5p	1.98	miR-30d-5p	1.03	miR-331-3p	2.53	miR-202-3p	1.052
11	miR-769-5p	8.65	miR-155-5p	2.06	miR-296-5p	1.077	miR-543	2.57	miR-1291	1.086
12	miR-373-3p	9.56	miR-886-5p	2.07	RNU44	1.125	miR-10b-3p	2.57	miR-331-3p	1.138
13	miR-1291	9.96	miR-193a-5p	2.07	miR-339-3p	1.163	miR-202-3p	2.58	miR-10b-3p	1.198
14	miR-543	10.04	miR-26a-5p	2.13	miR-769-5p	1.193	miR-1291	2.59	miR-543	1.21
15	miR-339-3p	10.29	miR-192-5p	2.13	miR-202-3p	1.224	miR-99b-5p	2.64	miR-345-5p	1.355
16	miR-99b-5p	12.45	miR-339-3p	2.14	miR-1291	1.273	miR-345-5p	2.67	miR-99b-5p	1.364
17	miR-211-5p	13.21	miR-331-3p	2.17	miR-328	1.337	miR-542-3p	2.78	miR-886-5p	1.652
18	miR-345-5p	15.23	miR-30d-5p	2.19	miR-720	1.394	miR-886-5p	2.84	miR-542-3p	1.692
19	miR-542-3p	16.03	miR-369-3p	2.19	miR-362-3p	1.444	miR-328	2.87	miR-373-3p	1.798
20	miR-886-5p	17.05	miR-335-3p	2.25	miR-1275	1.491	miR-373-3p	2.94	miR-362-3p	1.816
21	miR-25-3p	19.89	miR-10b-3p	2.27	miR-211-5p	1.54	miR-362-3p	2.96	miR-211-5p	1.827
22	miR-328	20.11	miR-543	2.3	miR-373-3p	1.607	miR-720	2.97	miR-328	1.832
23	miR-362-3p	21.34	miR-328	2.33	miR-886-5p	1.668	miR-211-5p	2.98	miR-720	2.024
24	miR-720	22.27	miR-19a-3p	2.36	miR-542-3p	1.718	miR-1275	3.05	miR-1275	2.139
25	miR-369-3p	24.39	miR-99b-5p	2.37	miR-190a	1.777	miR-25-3p	3.18	miR-494	2.212
26	miR-155-5p	24.5	miR-362-3p	2.44	miR-369-3p	1.853	miR-335-3p	3.22	miR-190a	2.216
27	miR-1275	24.83	miR-720	2.46	miR-25-3p	1.919	miR-190a	3.25	miR-369-3p	2.253
28	miR-335-3p	25.93	miR-190a	2.51	miR-494	1.979	miR-369-3p	3.26	miR-16-5p	2.366
29	miR-190a	26.48	miR-212-3p	2.57	miR-335-3p	2.037	miR-16-5p	3.28	miR-25-3p	2.37
30	miR-494	30.46	miR-659-3p	2.59	miR-323-3p	2.087	miR-494	3.28	miR-335-3p	2.427
31	miR-16-5p	30.85	miR-151a-3p	2.6	miR-16-5p	2.132	miR-323-3p	3.28	miR-323-3p	2.446
32	miR-323-3p	32.14	miR-345-5p	2.62	miR-155-5p	2.185	miR-155-5p	3.48	miR-155-5p	2.705
33	miR-212-3p	32.67	miR-1275	2.63	miR-301a-3p	2.236	miR-301a-3p	3.54	miR-301a-3p	2.709
34	miR-19a-3p	33.43	miR-340-3p	2.67	miR-212-3p	2.284	miR-212-3p	3.58	miR-212-3p	2.914
35	miR-151a-3p	34.44	miR-483-3p	2.72	miR-483-3p	2.334	miR-151a-3p	3.6	miR-483-3p	3.007
36	miR-301a-3p	34.63	miR-16-5p	2.8	miR-151a-3p	2.395	miR-483-3p	3.68	miR-151a-3p	3.035
37	miR-483-3p	35.25	miR-323-3p	2.82	miR-19a-3p	2.452	miR-19a-3p	3.71	miR-221-3p	3.095
38	miR-659-3p	36.52	miR-520b	2.96	miR-221-3p	2.506	miR-221-3p	3.76	miR-19a-3p	3.15
39	miR-221-3p	37.99	miR-221-3p	3.01	miR-659-3p	2.559	miR-659-3p	3.87	miR-659-3p	3.267
40	miR-340-3p	38.41	miR-301a-3p	3.17	miR-340-3p	2.613	miR-340-3p	3.98	miR-340-3p	3.529
41	miR-365	41.97	miR-494	3.31	miR-365	2.683	miR-365	4.17	miR-365	3.631
42	miR-758	42.25	miR-193b-5p	3.39	miR-758	2.753	miR-758	4.45	miR-758	4.031
43	miR-193b-5p	42.99	miR-758	3.67	miR-193b-5p	2.849	miR-193b-5p	5.18	miR-138-5p	4.816
44	miR-138-5p	43.75	miR-138-5p	4.01	miR-138-5p	2.956	miR-138-5p	5.26	miR-193b-5p	4.893
45	miR-520b	44.57	miR-365	4.15	miR-342-3p	3.061	miR-342-3p	5.28	miR-342-3p	4.979
46	miR-342-3p	45.25	miR-342-3p	4.7	miR-199a-3p	3.162	miR-199a-3p	5.45	miR-199a-3p	5.161
47	miR-199a-3p	46.25	miR-199a-3p	5.03	miR-520b	3.309	miR-520b	6.63	miR-520b	6.208

**Table A7:** miRNA stability ranking in all samples (ALL).

Ranking	Geomean		BestKeeper		geNorm		delta-Ct		NormFinder	
1	miR-26a-5p	1.86	miR-202-3p	1.04	miR-26a-5p | miR-331-3p	0.579	miR-26a-5p	2.3	miR-26a-5p	0.555
2	RNU44	3.31	miR-211-5p	1.06			RNU44	2.32	miR-26b-5p	0.56
3	miR-26b-5p	3.98	miR-1291	1.25	miR-26b-5p	0.714	miR-26b-5p	2.33	RNU44	0.591
4	miR-769-5p	4.68	RNU44	1.28	miR-296-5p	0.771	miR-769-5p	2.35	miR-769-5p	0.698
5	miR-331-3p	5.19	miR-769-5p	1.31	RNU44	0.858	miR-296-5p	2.4	miR-30d-5p	0.823
6	miR-296-5p	6.12	miR-373-3p	1.37	miR-769-5p	0.93	miR-30d-5p	2.4	miR-193a-5p	0.836
7	miR-30d-5p	6.59	miR-155-5p	1.51	miR-30d-5p	0.97	miR-331-3p	2.42	miR-296-5p	0.897
8	miR-193a-5p	7.44	miR-193a-5p	1.52	miR-193a-5p	1.019	miR-193a-5p	2.42	miR-331-3p	0.921
9	miR-1291	9.12	miR-30d-5p	1.57	miR-345-5p	1.09	miR-345-5p	2.48	miR-345-5p	0.98
10	miR-202-3p	9.41	miR-296-5p	1.59	miR-543	1.139	miR-543	2.55	miR-543	1.254
11	miR-211-5p	10.81	miR-542-3p	1.65	miR-10b-3p	1.207	miR-542-3p	2.58	miR-542-3p	1.434
12	miR-345-5p	10.99	miR-26a-5p	1.67	miR-323-3p	1.306	miR-1291	2.69	miR-1291	1.447
13	miR-543	11.58	miR-331-3p	1.69	miR-16-5p	1.375	miR-10b-3p	2.7	miR-10b-3p	1.551
14	miR-542-3p	11.68	miR-26b-5p	1.74	miR-542-3p	1.433	miR-16-5p	2.75	miR-369-3p	1.557
15	miR-373-3p	14.56	miR-369-3p	1.8	miR-190a	1.493	miR-323-3p	2.76	miR-16-5p	1.592
16	miR-10b-3p	15.37	miR-328	1.83	miR-1291	1.552	miR-328	2.78	miR-323-3p	1.625
17	miR-16-5p	15.47	miR-25-3p	1.85	miR-373-3p	1.605	miR-369-3p	2.8	miR-202-3p	1.656
18	miR-323-3p	16.21	miR-190a	1.86	miR-211-5p	1.648	miR-190a	2.81	miR-190a	1.665
19	miR-369-3p	16.55	miR-543	1.86	miR-25-3p	1.694	miR-211-5p	2.84	miR-328	1.671
20	miR-155-5p	17.27	miR-345-5p	1.89	miR-339-3p	1.735	miR-373-3p	2.85	miR-211-5p	1.702
21	miR-190a	17.43	miR-16-5p	1.92	miR-369-3p	1.774	miR-202-3p	2.86	miR-494	1.805
22	miR-328	18.29	miR-151a-3p	2.01	miR-202-3p	1.813	miR-25-3p	2.92	miR-373-3p	1.811
23	miR-25-3p	20.53	miR-339-3p	2.02	miR-328	1.857	miR-155-5p	2.93	miR-155-5p	1.893
24	miR-339-3p	22.69	miR-323-3p	2.09	miR-155-5p	1.892	miR-339-3p	2.94	miR-339-3p	1.953
25	miR-494	25.65	miR-340-3p	2.09	miR-494	1.933	miR-494	2.94	miR-25-3p	2.064
26	miR-151a-3p	25.87	miR-19a-3p	2.13	miR-335-3p	1.973	miR-151a-3p	3.09	miR-192-5p	2.114
27	miR-192-5p	28.41	miR-192-5p	2.16	miR-151a-3p	2.011	miR-335-3p	3.13	miR-335-3p	2.214
28	miR-335-3p	28.54	miR-520b	2.19	miR-340-3p	2.058	miR-1275	3.15	miR-1275	2.287
29	miR-340-3p	29.31	miR-1275	2.24	miR-19a-3p	2.104	miR-192-5p	3.17	miR-151a-3p	2.321
30	miR-1275	29.43	miR-10b-3p	2.26	miR-221-3p	2.144	miR-720	3.28	miR-720	2.476
31	miR-19a-3p	31	miR-720	2.26	miR-758	2.186	miR-340-3p	3.33	miR-362-3p	2.508
32	miR-720	31.21	miR-758	2.32	miR-192-5p	2.226	miR-362-3p	3.33	miR-301a-3p	2.62
33	miR-221-3p	32.71	miR-494	2.38	miR-1275	2.276	miR-301a-3p	3.38	miR-221-3p	2.674
34	miR-758	33.9	miR-221-3p	2.44	miR-720	2.324	miR-221-3p	3.4	miR-340-3p	2.683
35	miR-362-3p	34.11	miR-335-3p	2.49	miR-362-3p	2.369	miR-19a-3p	3.43	miR-19a-3p	2.819
36	miR-301a-3p	35.12	miR-886-5p	2.59	miR-301a-3p	2.412	miR-758	3.54	miR-483-3p	2.831
37	miR-483-3p	37.93	miR-659-3p	2.61	miR-483-3p	2.46	miR-483-3p	3.57	miR-758	2.889
38	miR-138-5p	38.75	miR-138-5p	2.65	miR-365	2.516	miR-365	3.69	miR-365	3.023
39	miR-365	39.42	miR-362-3p	2.69	miR-138-5p	2.574	miR-138-5p	3.91	miR-138-5p	3.156
40	miR-659-3p	39.47	miR-301a-3p	2.78	miR-659-3p	2.643	miR-659-3p	4.24	miR-99b-5p	3.645
41	miR-520b	39.97	miR-99b-5p	2.79	miR-342-3p	2.716	miR-342-3p	4.32	miR-659-3p	3.731
42	miR-99b-5p	41.24	miR-483-3p	2.8	miR-99b-5p	2.79	miR-99b-5p	4.32	miR-342-3p	3.774
43	miR-886-5p	41.61	miR-193b-5p	2.98	miR-193b-5p	2.865	miR-193b-5p	4.6	miR-886-5p	4.167
44	miR-342-3p	42.22	miR-365	3.01	miR-886-5p	2.946	miR-886-5p	4.74	miR-193b-5p	4.199
45	miR-193b-5p	43.25	miR-342-3p	3.28	miR-520b	3.031	miR-520b	4.89	miR-520b	4.31
46	miR-199a-3p	46.25	miR-212-3p	3.89	miR-199a-3p	3.121	miR-199a-3p	5.09	miR-199a-3p	4.736
47	miR-212-3p	46.75	miR-199a-3p	4.31	miR-212-3p	3.212	miR-212-3p	5.28	miR-212-3p	4.787
